# High Precision Mammography Lesion Identification From Imprecise Medical Annotations

**DOI:** 10.3389/fdata.2021.742779

**Published:** 2021-12-03

**Authors:** Ulzee An, Ankit Bhardwaj, Khader Shameer, Lakshminarayanan Subramanian

**Affiliations:** ^1^ Department of Computer Science, University of California, Los Angeles, Los Angeles, CA, United States; ^2^ Department of Computer Science, Courant Institute of Mathematical Sciences, New York University, New York, NY, United States; ^3^ Northwell Health, New York, NY, United States; ^4^ Department of Population Health, NYU Grossman School of Medicine, New York University, New York, NY, United States

**Keywords:** oncology, breast cancer, computer vision, digital health, computational diagnosis, big data and analytics

## Abstract

Breast cancer screening using Mammography serves as the earliest defense against breast cancer, revealing anomalous tissue years before it can be detected through physical screening. Despite the use of high resolution radiography, the presence of densely overlapping patterns challenges the consistency of human-driven diagnosis and drives interest in leveraging state-of-art localization ability of deep convolutional neural networks (DCNN). The growing availability of digitized clinical archives enables the training of deep segmentation models, but training using the most widely available form of coarse hand-drawn annotations works against learning the precise boundary of cancerous tissue in evaluation, while producing results that are more aligned with the annotations rather than the underlying lesions. The expense of collecting high quality pixel-level data in the field of medical science makes this even more difficult. To surmount this fundamental challenge, we propose LatentCADx, a deep learning segmentation model capable of precisely annotating cancer lesions underlying hand-drawn annotations, which we procedurally obtain using joint classification training and a strict segmentation penalty. We demonstrate the capability of LatentCADx on a publicly available dataset of 2,620 Mammogram case files, where LatentCADx obtains classification ROC of 0.97, AP of 0.87, and segmentation AP of 0.75 (IOU = 0.5), giving comparable or better performance than other models. Qualitative and precision evaluation of LatentCADx annotations on validation samples reveals that LatentCADx increases the specificity of segmentations beyond that of existing models trained on hand-drawn annotations, with pixel level specificity reaching a staggering value of 0.90. It also obtains sharp boundary around lesions unlike other methods, reducing the confused pixels in the output by more than 60*%*.

## 1 Introduction

Locating medical abnormalities have historically confounded human-driven clinical procedures. Over the history of radiology, the *Breast Imaging Reporting and Data System* (BI-RADs) (Rao et al., 2016) has outlined the procedure of identifying and annotating these critical lesions. However retrospective studies on the effectiveness of radiology assessment have reported that cancer lesions could be identified retroactively in past X-rays for more than 30*%* of patients who were later diagnosed at advanced stages (Solveig, 2012). Today annotation tools for medical images take on an assistive role, but the need for performant and end-to-end diagnosis models are felt strongly in this context (Badgeley et al., 2019).

Deep neural nets are a strong candidate in automating the diagnosis of medical images as they demonstrate state-of-art performance in the domain of computer vision. To this end, datasets of annotated medical imaging have been available for decades since such as DDSM (Heath et al., 1998) which was compiled in 1997. Deep neural-net based models have demonstrated expert performance in classification problems for general objects given large datasets which have become available (Lin et al., 2014), however we find that they are not immune to the presence of mislabelled examples presented as ground-truth which is characteristic of coarsely annotated data. Here, segmentation models experience a fundamental challenge with hand-drawn annotations which are encountered when existing clinical archives are digitized and made available for analysis. It is reasonable to expect this given that these archives were meant to be consumed by human experts rather than computational models. As such, hand drawn boundaries indicate a general area of abnormality and often do not separate true-positive features from the image with pixel-level accuracy. Treating loose annotations as direct annotations inevitably introduces a margin of uncertainty characterized by features which may or may not belong to the target class, and we assess that the most widely used segmentation models would not be immune to this effect. The massive cost for collecting pixel-level accurate data in the medical domain greatly hampers the utility of these models.

To surmount these challenges we define **LatentCADx**, a modular joint classification-segmentation model which is procedurally trained in the presence of imperfect segmentations. The joint objective leverage the property that features driving high classification accuracy should be consistent between multiple objectives containing ground-truth. Furthermore in the context of breast cancer tissue assessment, we impose a strict penalty in segmentation training such that predictions do not egress hand-drawn annotations. We therefore subject segmentations to the circumscribing boundary of the annotations and underlying features responsible for classification with high accuracy.

In our results, LatentCADx improves upon both classification and segmentation performance, and predicts segmentations which are more expressive and specific than possible with existing approaches. It should be noted that other existing approaches are fully capable of segmenting complex lesions with high accuracy as long as they are provided with high-quality annotated data. Thus, our focus while developing LatentCADx has been on circumventing the inaccurate annotations and identifying latent lesions rather than improving segmentation performance measured based on the said annotations. The main contributions of our work are as follows:

• Using joint classification and segmentation architecture and weakly-supervised loss function, we obtain LatentCADx model that performs on par or better than other segmentation models.
• LatentCADx provably reduces the uncertainty in the boundary of segmentation results.
• LatentCADx unearths underlying lesions from coarse hand-drawn annotations and produces high precision segmentations.

## 2 Related Works

### 2.1 Feature Engineering and Matching

Manually engineered features have enabled several approaches to automatic detection with no learning. Regions of interest are detected with filters which highlight domain-specific tissue morphology found in Mammography (Eltonsy et al., 2007; Song et al., 2009; He et al., 2010). However, the process of crafting individual filters requires prior domain knowledge. For example, specific methods have been developed to detect spiculations (Kobatake and Yoshinaga, 1996; Muralidhar et al., 2010) and further work would be required for additional categories of lesions.

### 2.2 Convolutional Neural Nets

Convolutional Neural Nets (LeCun and Bengio, 1998) rely on learning the features relevant to a classification task during optimization without the need for feature crafting. The motivation for CNNs in medical imaging arises from their ability to obtain state-of-art accuracies in general object classification tasks (Krizhevsky et al., 2012; He et al., 2015; Szegedy et al., 2015). Their convolutional architectures can be adapted by design to predict the location of classes, a driving feature of many proposed segmentation models such as SegNet (Badrinarayanan et al., 2017), U-Net (Ronneberger et al., 2015), FCN (Long et al., 2015), and Mask-RCNN (He et al., 2017).

Several recent works have relied on variants of region proposal networks [RCNN (Ren et al., 2015)] for histology image segmentation (Jeremiah, 2018), brain MRI segmentation (Akkus et al., 2017), and lesion annotations in mammograms (Ribli et al., 2018). These mammogram studies find that applying existing deep architectures can already yield high classification performance. However, we note in these prior works that segmentation were not attempted as pixelwise ground-truth annotations are generally not available for mammograms.

(Abdelhafiz et al., 2020) outlines a detailed benchmark of applying U-Net directly to mammogram annotations. In this study, Mass lesions are specifically examined which we find are types of lesions which most closely adheres to clinical annotations. In reality lesions span a variety of categories and our testing spans both Mass and Calcifications, the latter of which have the most coarse annotations. We assess that an accurately annotated dataset of Calcifications would improve the reliability of segmentation models as demonstrated in (Wang et al., 2016), but such datasets are few and also not publicly available. We note one increasing difficulty in comparing to former works which leverage the INBreast mammogram dataset (Moreira et al., 2012) as the dataset is no longer publicly available due to data privacy laws.

### 2.3 Deep Mammography

Recent efforts have focused on addressing challenges specific to mammography through neural-net design. The extreme resolution of mammogram imaging data has been a point of technical difficulty for deep learning. To this end (Shen et al., 2019) describes a procedural method of training convolutional nets on local patches of tissue, then expanding training to full scans of mammograms to improve classification performance (Lotter et al., 2021). further extends the idea of procedural training on tissue patches to classifying mammograms which are obtained as 3D volumes. In (Wu et al., 2020) the authors introduce a novel architecture in combining the left, right, and scans of varying viewpoints for a patient-level classification. Comparatively, our work focuses on challenges with predicting lesion annotations which would be apparent in any such datasets of mammograms. We expect our contribution could improve such procedurally trained models when attempting full-scan or patient-level diagnoses.

### 2.4 Joint Classification and Segmentation

The idea of applying joint classification and segmentation models to medical images has been explored before in (Chakravarty and Sivswamy, 2018; Wang et al., 2019; Heker and Greenspan, 2020; Ryu et al., 2021) among others in different ways. But the underlying motivation and approach used in the above papers is different from what we have tried to accomplish in our work. The multi-task learning framework has mostly been used to ensure generalization of deep learning models which is known to benefit the performance on both tasks. We, on the other hand, have used the same generalization feature to overcome the quality of hand-drawn annotations on the segmentation task. Adding on, the constraint objective used in training, we obtain sharper segmentation results as compared to other methods. We could not find examples of this approach on mammography datasets for comparison.

## 3 Dataset and Calibration

**FIGURE 1:**
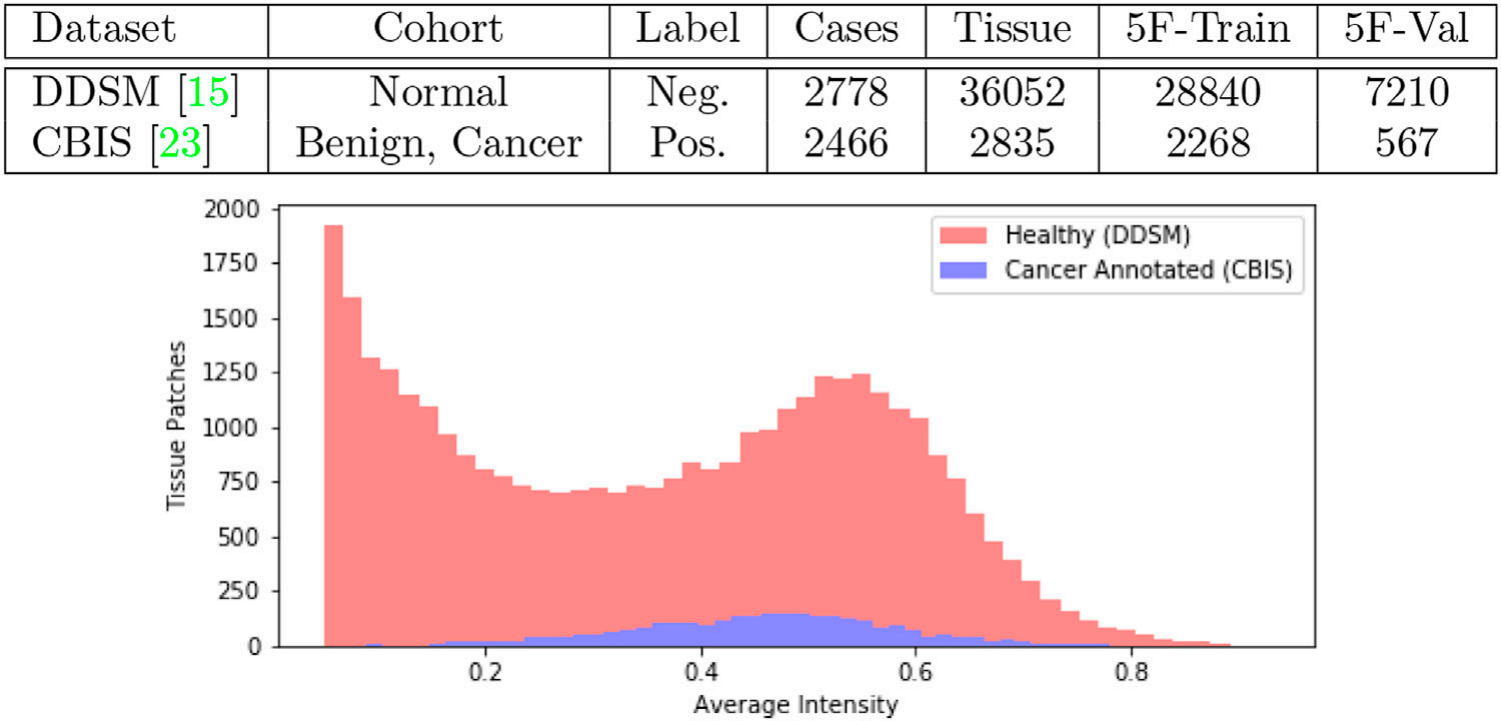
Joint DDSM-CBIS Dataset. We gather positive and negative cases of cancerous tissue from DDSM (Heath et al., 1998), the largest publicly available source of mammogram data, and CBIS-DDSM (Lee et al., 2006) which improves the accuracy of annotations in the original dataset. In total, we sample 38,887 patches of tissue from the mammograms. We ensure that both datasets are calibrated identically and organized into 5-Folds (5F).

We obtained mammogram data from the *Digital Database for Screening Mammography* (DDSM) which is a public repository containing cases of healthy individuals, individuals with benign breast cancer and individuals with malignant breast cancer (Heath et al., 1998). An updated study cataloged by *The Cancer Imaging Archive* under the name *Curated Breast Imaging Subset of DDSM* (CBIS-DDSM) revised every cancer annotation in DDSM and was published in 2017 (Lee et al., 2006). In Figure 1 we tabulate the total number of samples we obtained from the datasets. In our work, we collect samples from individuals with malignant lesions and healthy individuals.

To obtain the most up-to-date annotations, positive samples were collected from the CBIS dataset while negative samples were collected from the healthy cohort in the original DDSM dataset. For the negative samples acquired from DDSM, we followed the instructions in the data publication to convert raw mammogram data where intensity per pixel is digitized in 256^2^ integer scale to a linear floating optical density scale between 0 ∼ 3 (Heath et al., 1998). The optical density scale was then normalized between 0 ∼ 1 to match the CBIS mammograms which were already processed with the calibration and normalization. Before normalization, we further clipped optical densities below 0.05 to 0 and above 3.0 to 3.0 as outlined by CBIS. All imaging data were therefore mapped to a scale of 0 ∼ 1 per pixel for all downstream tasks. Mammogram scans which were originally at a resolution of 4,000 × 6,000 pixels, were padded to the maximum resolution, and then downsampled × 0.25 to 1,000 × 1,500. Finally, for positive images, we obtained the center-of-mass for region of interest in CBIS annotations, from which we sampled 256 × 256 resolution patches of tissue. For negative images, we used a sliding window to generate patches of the same size. In total we collected 2,835 positive samples and 36,052 negative samples.

At inference time, we can use the sliding window to convert test images into patches and aggregate the predictions from different patches to make image-level predictions. Under this procedure, it is evident that the number of negative patches encountered would be many times greater than the number of positive patches. To simulate this behaviour, we chose to keep such imbalance in our training data as well.

As a diagnostic procedure, we confirmed that the average intensities of the mammograms approximately align across the two datasets (Figure 1). During training, the image data underwent augmentations such as random rotation (±15^°^), random crop, random noise so that the data bias was expected to be negligible.

## 4 Methods

### 4.1 Feature Extraction

**FIGURE 2:**
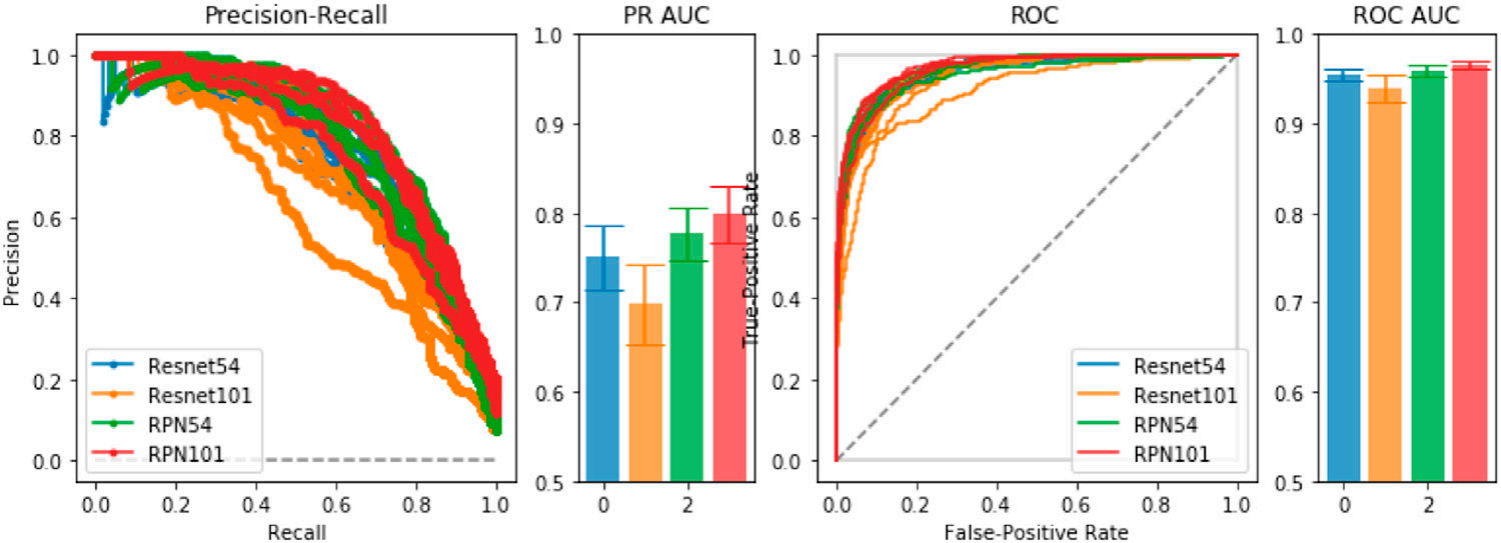
Model Selection. We compared ResNet-54, ResNet-101 and a joint prediction and region-proposal model architecture (RPN) based on the ResNet model. Under Precision-Recall and the Receiver Operating Characteristic, the joint predictors RPN54 and RPN101 both significantly outperformed their baseline counterparts with significance. We chose RPN54 as the feature extractor for the full LatentCADx model.

To empirically diagnose training and model choice, we performed 5-fold validation between several ResNet-based architectures. In the comparisons, we include a Region-Proposal Network (RPN) based on the ResNet architecture from which we obtain initial pre-trained weights for training the full LatentCADx model. In Figure 2, we found that the 54-layer ResNet model (ResNet54) would ideally fit the dataset and obtained higher Precision-Recall (PR) of 0.75 over ResNet101 which obtained 0.70 with significance (*p* < 0.005). Proceeding with ResNet54, we developed the RPN starting from the feature map (8 × 8 × 2048) and ending with an output of 8 × 8 × 2. The RPN was therefore capable of classifying the tissue images in addition to predicting the coarse location of lesions in each region. The RPN models improved incrementally in performance over their standalone ResNet counterparts, where RPN54 reached up to 0.80 in AP. Between RPN54 and RPN101, the former model had far fewer parameters, yet their difference in performance was not significant. Using ROCAUC, all models obtained a high metric which can be attributed to high imbalance in the data between positive and negative samples. Performance by this measure was similar ( ≥0.95 ) across the ResNet-based models and the differences were significant.

The RPN54 classifier obtained from joint training was leveraged as a feature extractor for the LatentCADx model. All ResNet-based models were trained for 50 epochs with step-wise learning rate progressing from 0.001 and decreasing by half every ten epochs on a single Nvidia P40 GPU using the SGD optimizer.

### 4.2 LatentCADx Architecture

#### 4.2.1 Convolution Head

The LatentCADx architecture was built upon ResNet convolution head such that we could perform weight transfer from a pre-trained ResNet model with high classification accuracy. We initialized these layers using the weights from 54-layer region-proposal model (RPN54), which was determined to perform the best with 0.80 in PR and 0.95 ROC AUC. To maintain these weights through training LatentCADx, we scheduled training such that for the first 15 epochs, all weights transferred from RPN54 were locked such that only the output head leading to the final 256 × 256 segmentation image was trained. After the 15 epochs, the layers were unfrozen such that the convolution head could be improved. In this stage, both classification and segmentation outputs were trained, while the region-proposal output was discarded.

#### 4.2.2 Unpooling of Activated Features

**FIGURE 3:**
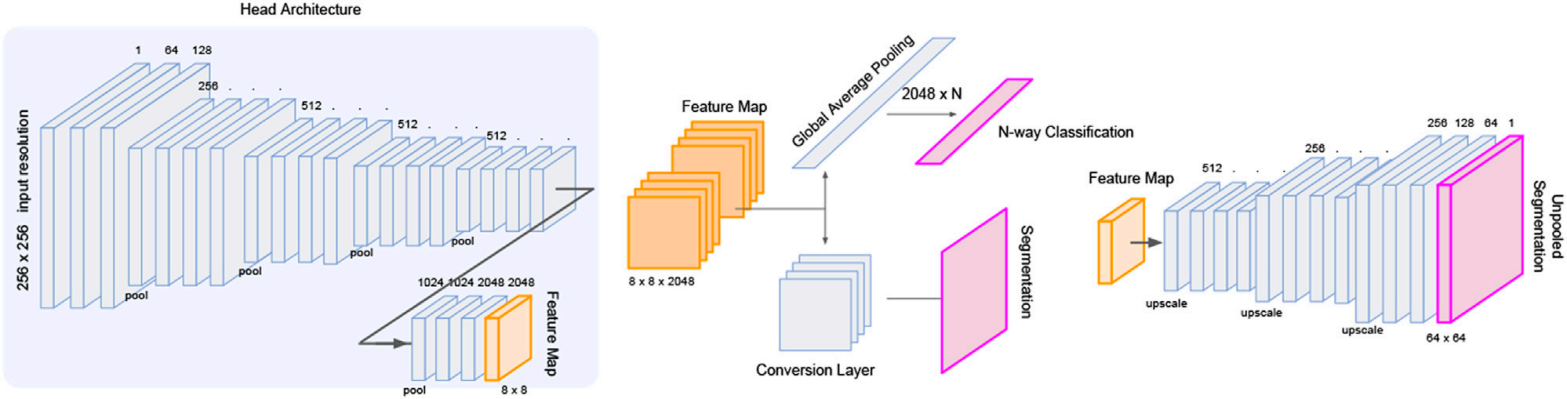
LatentCADx Architecture. LatentCADx consist of a modular architecture which allows weight transfer and selective training of layers and prediction outputs. We outline a procedural approach to training LatentCADx such that features driving high classification accuracy are consistent with final segmentation image (diagram created via Google Slides https://slides.google.com).

Several architectures exist to transition deep feature map in latent space to an interpretable image such as unpooling and deconvolution, further modifiable with skip connections (Ronneberger et al., 2015) or pyramid feature mapping (Lin et al., 2017). Skipping architectures allow information to carry over from initial layers of the neural net before down-convolutions, and factor in as additional activations in the up-convolutions leading to the segmentation prediction. We implement a series of deconvolution blocks which takes as input the forward evaluated features and skipped features from the initial ResNet layers. From the deepest feature map of 8 × 8 × 2048, each deconvolution increases the sizes of the image dimension by a factor of 2 while reducing the number of latent dimensions. The final output of the deconvolution head arrives at a dimension of 256 × 256 × 1 which is interpreted as a segmentation image. We visualize the architecture of the full model in Figure 3.

### 4.3 Weakly-Supervised Objective

Separate treatment is given to segmentation areas which egress outside or remain inside the clinical hand-drawn boundary. We build upon the combination of Categorical Cross-Entropy (*CrossEnt*) (Rubinstein, 1999) and Mean-Square Error (*MSE*) (Makhzani et al., 2015) losses for the objective of simultaneous classification and localization (Ross, 2015; He et al., 2017). Hyperparameters *α* and *β* can control the prioritization of either part of the objective during training.

$$CrossEnt\left(y,\hat{y}\right)=-\hat{y}\log\left(y\right)+\left(1-\hat{y}\right)\log\left(1-y\right)$$ (1)

$$MSE\left(M,\hat{M}\right)=\frac{1}{N}\overset{N}{\underset{i=1}{\sum }}{\left(M_{i}-\hat{M_{i}}\right)}^{2}$$ (2)

$$L\left(y,\hat{y},M,\hat{M}\right)=\alpha *CrossEnt\left(y,\hat{y}\right)+\beta^{*}MSE\left(M,\hat{M}\right)$$ (3)

We define $\hat{M}$ as the ground-truth segmentation mask, which in our context were hand-drawn annotations of lesions converted to binary masks in tissue samples.

Ideally the mask is assumed to be reasonably semantically consistent with the target subject in the images for the best segmentation results. We note once more that for the mammography task that the annotated boundaries are far from pixel-perfect. To overcome this challenge, we first relied on the convolution features driving high accuracy in binary prediction by weight transfer. Then in segmentation training, we treat the area outside the segmentation boundary *oob* and inside the boundary *inb* separately, allowing greater flexibility for mask loss $L_{\hat{M}}$ .

$$L_{\hat{M}}∼\beta *L_{inb}+\gamma *L_{oob}$$ (4)

Element-wise product with the inverted mask $1-\hat{M}={\hat{M}}^{\prime }$ with the prediction can indicate the *oob* positions which egress outside the annotated boundary. As we tolerate no predictions in *oob*, a heavy loss is incurred for such predictions. The penalty for this region is applicable to $N_{oob}=\sum_{i=1}^{N}{\hat{M}}_{i}^{\prime }$ positions and can be expressed as:

$$L_{oob}=\frac{1}{N_{oob}}\overset{N}{\underset{i=1}{\sum }}{\hat{M}}_{i}^{\prime }{\left(M_{i}-0\right)}^{2}=\frac{1}{N_{oob}}\overset{N}{\underset{i=1}{\sum }}{\hat{M}}_{i}^{\prime }M_{i}^{2}$$ (5)

Note that the sum part ( $\sum_{i=1}^{N}{\hat{M}}_{i}^{\prime }M_{i}^{2}$ ) essentially represents the sum over all elements in ${\hat{M}}^{\prime }$ with *N*_*oob*_ elements. Thus, *L*_*oob*_ is an MSE loss over ${\hat{M}}^{\prime }$ . Similarly, the region in-bounds *inb* can be indicated element-wise by the annotation mask *M*. Due to the joint architecture of LatentCADx, segmentation predictions within *inb* are driven by features which also contribute to the accuracy of $\hat{y}$ . The valid area within bounds can be tallied as $N_{inb}=\sum_{i=1}^{N}{\hat{M}}_{i}$ .

$$L_{inb}=\frac{1}{N_{inb}}\overset{N}{\underset{i=1}{\sum }}{\hat{M}}_{i}{\left({\hat{M}}_{i}-M_{i}\right)}^{2}$$ (6)

In the presence of unreliable ground-truth annotations for *inb*, we regularize the overall parameter space through pre-training, scheduling and prioritization of objectives. We prioritize the different objectives using hyperparameters *α*, *β* and *γ*, expressing the full objective:

$$\begin{array}{ll} L\left(y,\hat{y},M,\hat{M}\right) & =\alpha *CrossEnt\left(y,\hat{y}\right)\\ & +\beta *\left(\frac{1}{N_{inb}}\overset{N}{\underset{i=1}{\sum }}{\hat{M}}_{i}{\left({\hat{M}}_{i}-M_{i}\right)}^{2}\right)\\ & +\gamma *\left(\frac{1}{N_{oob}}\overset{N}{\underset{i=1}{\sum }}{\hat{M}}_{i}^{\prime }M_{i}^{2}\right) \end{array}$$ (7)

As the model should not compromise classification accuracy, nor produce segmentations outside the annotation boundary, the weighting of the hyperparameters were prioritized as *β* < *γ* < *α*.

Training of the model was performed using one Nvidia P100 instance over 100 epochs with early termination. We chose the SGD optimizer with initial training weight of 0.001 and scheduled a decrease in learning rate by a factor of half every 10 epochs. A learning schedule was implemented such that for the first 15 epochs, the *inb* and *oob* weights *β* and *γ* were unweighted and equal to 1. After 15 epochs, all layers of LatentCADx were trained which included loss from the classification output. For the full training stage, the loss weights were configured with *α* = 2, *β* = 1 and *γ* = 0.5. For all training procedures, we used a batch size of 32 images per batch and allows early termination when validation loss did not change within 1 × 10^−6^. The convergence of all ResNet-based models and the final LatentCADx model is shown in the Supplementary Materials (Supplementary Figures S2, S3).

## 5 Results

### 5.1 Performance Evaluation With Imprecise Annotations

**FIGURE 4:**
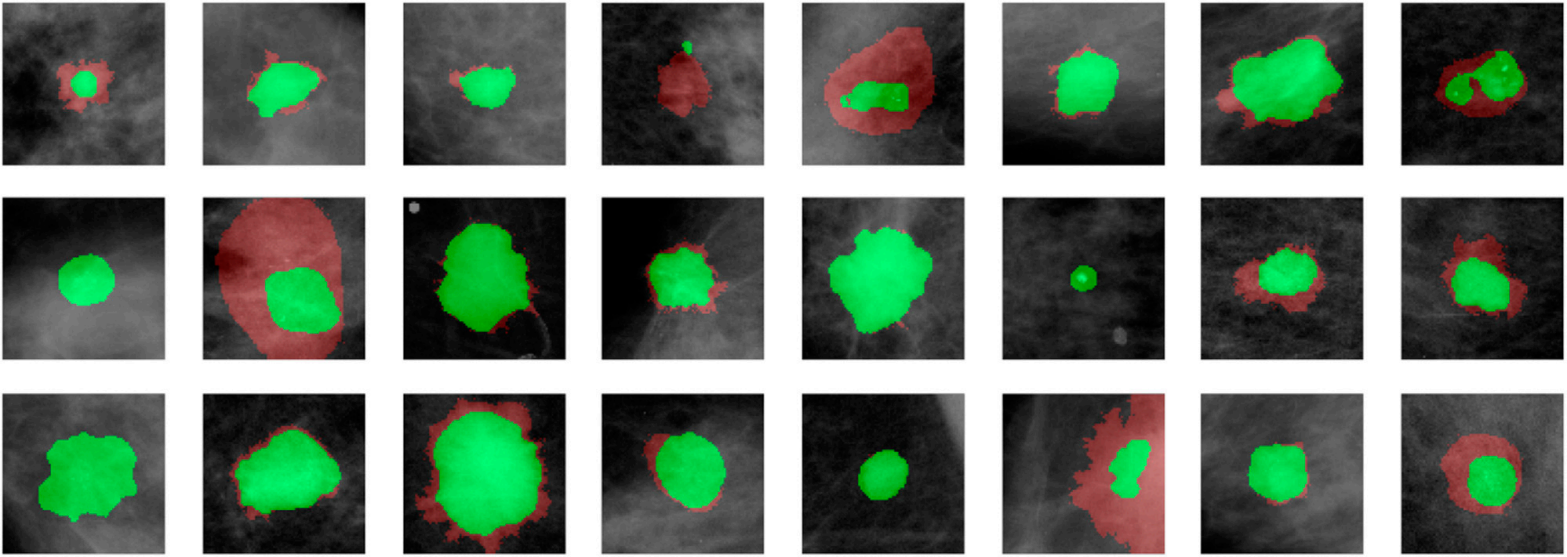
LatentCADx Segmentation. Clinical annotations are denoted in red and LatentCADx segmentations are overlayed in green. The weakly-supervised model constrained with multiple objectives predicted segmentation labels with higher detail than the annotations viewed in training. Predictions are visualized from validation samples which were not observed by the model until evaluation (Mammograms obtained from (Lee et al., 2006); CC BY open access license).

**FIGURE 5:**
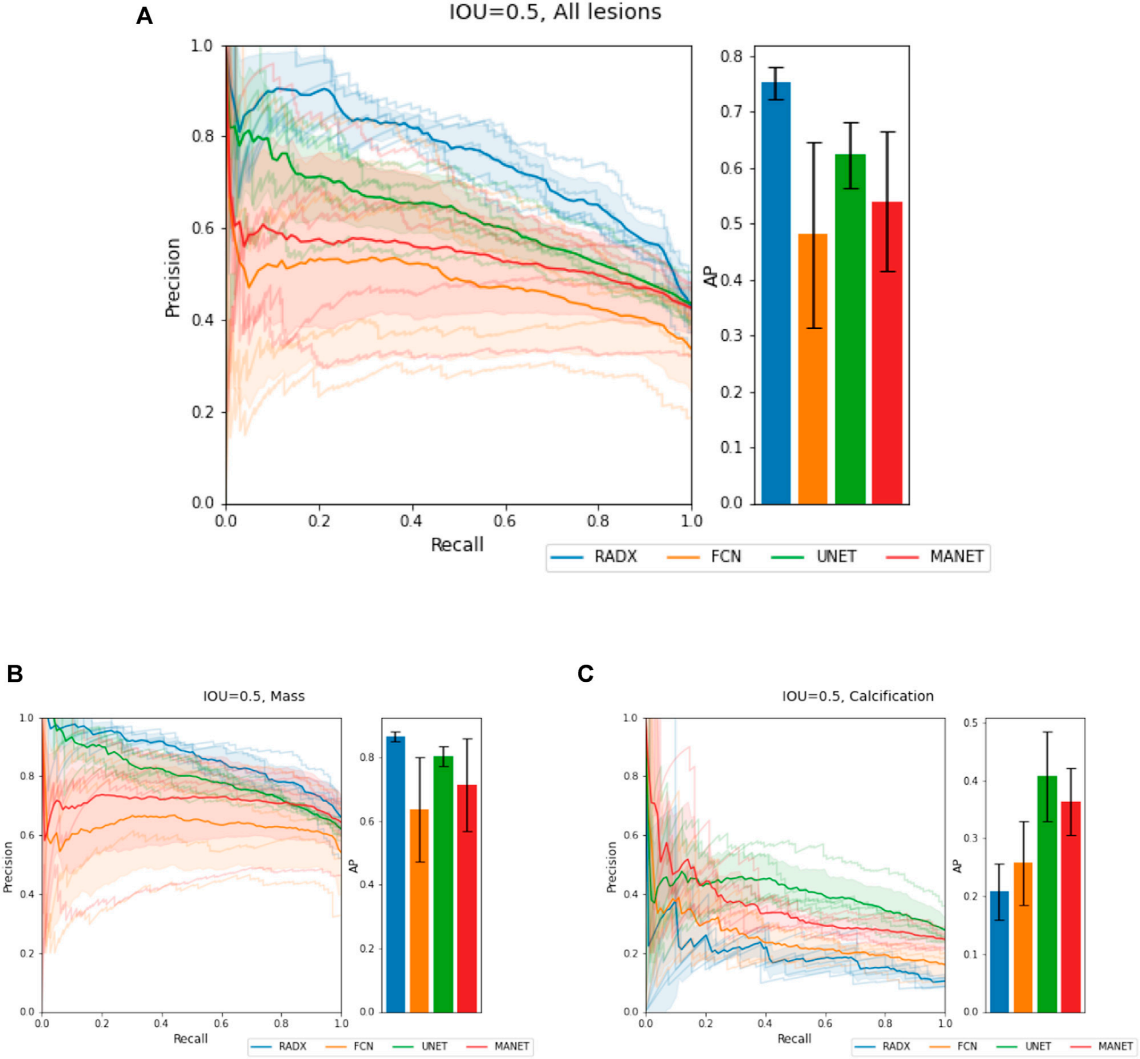
Segmentation Characteristics. Segmentation performance of LatentCADx (LCX) was measured using Average Precision (AP) based on precision-recall, for fixed Intersection Over Union (IOU) thresholds. **(A)** For IOU = 0.5 on all types of lesions, LatentCADx obtained the highest AP, followed by comparable segmentation models U-Net and FCN. Further breakdown on performance is given by separating predictions for lesions by **(B)** Mass, where LatentCADx also obtained the highest AP and **(C)** Calcifications.

We demonstrate quantitatively and qualitatively that LatentCADx detects underlying features of lesions, improving the quality of lesion segmentations. Several examples of annotations were visualized for which LatentCADx segmentations captured underlying lesions with greater detail within hand-drawn annotations (Figure 4). We quantified and compared segmentation performance of our model based on Average Precision (AP) on specific IOU thresholds of 0.3, 0.5, and 0.7 as defined in the COCO (Lin et al., 2014) and PASCAL VOC (Everingham et al., 2015) challenges. In comparisons with U-Net (Ronneberger et al., 2015) [implemented using (Buda et al., 2019)’s official implementation], Fully Convolutional Networks (FCN) (Long et al., 2015), and Multi-Scale Attention Network (MANET) (Fan et al., 2020) [implemented using (Yakubovskiy, 2020)] (Figure 5), we found LatentCADx demonstrated the best overall AP score. The performance of AP = 0.75 was significantly better the second best model U-Net (AP = 0.62) on IOU = 0.5 threshold (Table 1). Performance was also separately measured for test samples categorized as Mass and Calcification type lesions, in which LatentCADx also obtained highest AP for Mass lesions. For Calcification lesions, U-Net and MANET demonstrated the highest performance.

**TABLE 1:** Segmentation Performance (based on imprecise annotations) Average Precision (AP) of segmentation predictions were measured for ground truth annotations at varying Intersection Over Union (IOU) thresholds 0.3, 0.5 and 0.7 for LatentCADx (LCX) and three comparable approaches. Segmentation performance was further examined by binning APs by lesions annotated as Mass (M) and Calcification (C).

Lesion	M & C			Mass			Calcification		
IOU	0.3	0.5	0.7	0.3	0.5	0.7	0.3	0.5	0.7
U-Net (Ronneberger et al., 2015)	0.84	0.62	0.37	0.94	0.80	0.51	**0.73**	**0.41**	0.19
FCN (Long et al., 2015)	0.69	0.48	0.26	0.86	0.64	0.36	0.48	0.26	0.05
LCX	**0.86**	**0.75**	**0.48**	**0.97**	**0.86**	**0.53**	0.41	0.21	0.15
MANET (Fan et al., 2020)	0.72	0.54	0.29	0.86	0.71	0.41	0.58	0.36	**0.19**

Bolded numbers indicate highest score obtained under each metric.

We note that the annotations taken as ground truth for calculation of these metrics are imprecise and thus, these metrics don’t accurately measure performance. This problem persists with other commonly used metrics like average IOU scores and DICE scores as well. Thus, in the next subsection, we introduce new metrics that give us more insights into the performance of these models and are more suited to the task at hand, and demonstrate the superiority of our method.

We also note that Calcification annotations rarely adhere to the underlying lesion area as compared to Mass annotations. The orthogonal performance between Mass and Calcifications lesions likely reflects the coarseness of Calcification annotations and the conservative segmentations for smaller underlying lesions by LatentCADx. We justify this claim in the next subsection.

We further access the performance of LatentCADx predictions by looking at the binary classification performance. In Table 2, we compared our classification results to baseline models VGG19 (Simonyan and Zisserman, 2014) and ResNet (He et al., 2015). In 5-fold testing we observed that using the LatentCADx features allowed the classifier to reach a final performance which was overall best in F1-Score, Precision-Recall, AP, and Receiver Operating Characteristic (ROC). Most notably, the LatentCADx-based classifier obtained 0.87 AP improving with signifiance upon 0.81 AP of the unmodified ResNet trained from scratch (*p* < 0.005). As the dataset was imbalanced due to higher number of healthy tissues than annotated tissues, there was smaller differences in ROC performance yet LatentCADx also obtained the highest metric of 0.97 ROCAUC.

**TABLE 2:** Classification Performance. We compared the classification accuracy of VGG19, standalone ResNet-54, and the final LatentCADx model as a classifier. Through weight transfer and additional joint training for segmentation under the LatentCADx objective, we observed an increase in classification performance of the final LatentCADx model.

Model	F1 score	Precision	Recall	AP	ROC
VGG19 (Simonyan and Zisserman, 2014)	0.75	0.84	0.68	0.83	0.95
ResNet54 (He et al., 2015)	0.74	0.80	0.68	0.81	0.95
LCX	**0.77**	**0.86**	**0.70**	**0.87**	**0.97**

Bolded numbers indicate highest score obtained under each metric.

Finally, we explored the necessity of procedural training in obtaining the LatentCADx model by training the same architecture from scratch with no feature transfer step. This alternative to LatentCADx was trained through random initialization and from scratch while using an identical architecture as the final LatentCADx model. Forgoing all procedural steps, the same architecture obtained 0.81 AP which was in line with the performance of unmodified ResNet, and could not reach the same performance as our final model (Supplementary Figure S1).

### 5.2 Precision Evaluation

In an overwhelming majority of hand-drawn annotations, the annotations circumscribe the lesion completely, leaving extra space between the underlying lesion and annotation boundary that captures healthy tissue. For simplification, we will ignore the minority cases where this is not true and the annotations don’t circumscribe the lesions.

Given this, IOU between prediction mask and annotations is not a valid metric to judge the performance of a segmentation model. Thus, we split IOU into two new metrics, namely **IOP** (Intersection over Prediction) and **IOA** (Intersection over Annotation) to reveal more details about the performance. As the name suggests,

$$IOP=\frac{Prediction\cap Annotation}{Prediction}$$ (8)

$$IOA=\frac{Prediction\cap Annotation}{Annotation}$$ (9)

IOP and IOA can also be understood as the pixel level specificity and sensitivity of the predictions. For the situation when the Annotation mask is known to imprecisely circumscribe the actual underlying lesion, any model that accurately predicts the underlying lesions will have high IOP and low IOA. On the other hand, the models that get confused by the circumscribing annotations will have higher IOA but lower IOP. Now, we look at the average IOA and IOP values for different models on the validation set.

In Table 3, we see that LatentCADx predictions have overwhelmingly and uniquely high mean IOP across all types of lesions. Our predictions score an IOP of 0.88 for Calcifications and 0.92 IOP for Mass, in contrast to U-Net with the next best IOP of 0.66 for both types of calcifications. In addition to high IOP, LatentCADx predictions also obtain the lowest mean IOA of 0.28 for all lesions and 0.13 for Calcifications specifically. No other models obtained similar IOA, where the next-best overall IOA of 0.44 was obtained using FCN. Thus, we demonstrate that LatentCADx predictions are majorly intersecting with the annotations but generally smaller in size. Given circumscribing annotations, we expect the unobserved underlying lesions to have a profile which is consistent with our findings using IOP and IOA. Considering that segmentation and classification performance using LatentCADx are comparable or better than other approaches, we claim that LatentCADx is likely targeting underlying lesions. Simultaneously, our result points to the deficiency in annotation quality for calcification cases, which was observed in qualitative evaluation.

**TABLE 3:** Intersection Performance. Mean intersection over union (IOU), prediction (IOP) and annotation (IOA) for LatentCADx and other models. Predicted pixel values of all models was normalized to 0–1 range and were threshold at 0.5. Intersection performance was further examined by binning the values by lesions annotated as Mass (M) and Calcification (C). LatentCADx has the maximum mean IOP and minimum mean IOA values.

Lesion	M & C			Mass			Calcification		
Metrics	IOU	IOP	IOA	IOU	IOP	IOA	IOU	IOP	IOA
U-Net (Ronneberger et al., 2015)	0.41	0.66	0.62	0.49	0.69	0.68	0.34	0.63	0.57
FCN (Long et al., 2015)	0.28	0.64	0.44	0.37	0.69	0.51	0.21	0.61	0.37
LCX	0.25	**0.90**	**0.28**	0.42	**0.92**	**0.44**	0.11	**0.88**	**0.13**
MANET (Fan et al., 2020)	0.40	0.66	0.62	0.50	0.69	0.70	0.31	0.62	0.55

Bolded numbers indicate highest score obtained under each metric.

**FIGURE 6:**
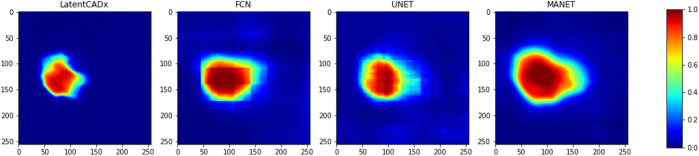
Confused Pixels. We find that all model outputs form a gradually decreasing intensity value as we leave the location of predicted lesion.

To further distinguish LatentCADx from the capabilities of existing methods, we seek to quantify the uncertainty in segmentation prediction in the boundary of lesions. To capture the uncertainty of this prediction boundary, we look at the pixel level probabilities in the predictions of different models. For each model, we observe varying degrees of confusion in the segmentation prediction, which is visualized in Figure 6.

As most annotations do not perfectly separate healthy tissue from the lesion areas, all models exhibit a boundary of uncertainty approaching the annotation boundary. This leads to large bands of uncertain pixels around the predicted lesions. To capture the size of this band, we define a new metric called **confused pixels**. In practice, we quantified confused pixels in the model predictions by thresholding the pixel values between 0.5 − *ϵ* and 0.5 + *ϵ* for some suitable value of *ϵ*. For *ϵ* = 0.2, we calculated the average number of confused pixels in the model outputs on the validation set and tabulated our results in Table 4. We observed a clear reduction in the mean number of confused pixels in LatentCADx. For all types of lesions, confused pixels were reduced × 2.5 fold in comparison to FCN, which was the next best model under this metric. We therefore assessed that our approach drastically reduces the uncertainty in the prediction boundary.

**TABLE 4:** Confused Pixels. We find out the average number of pixels in the predictions of different models such that their values lie between 0.3 and 0.7. We claim that these are confused pixels where the model is unsure of classifying them between healthy and pathological.

Metric: Mean number of confused pixels			
Lesion	M & C	Mass	Calcification
U-Net (Ronneberger et al., 2015)	6,878.00	5,337.25	8,186.59
FCN (Long et al., 2015)	4,335.97	4,127.27	4,513.22
LCX	**1,677.55**	**1811.41**	**1,563.86**
MANET (Fan et al., 2020)	9,238.39	7,322.93	10,865.23

Bolded numbers indicate highest score obtained under each metric.

Another look at Table 4 shows that for methods that take the annotations as ground truth labels, the mean number of confused pixels shows a marked increase from mass to calcification bracket. This is another indication that calcification annotations in the dataset are of poorer quality. On the other hand, our method in-fact decreases the number of confused pixels on calcification cases suggesting that our model is able to hone in on lesions with high precision.

### 5.3 Qualitative Evaluation

#### 5.3.1 Interpretable Segmentations

**FIGURE 7:**
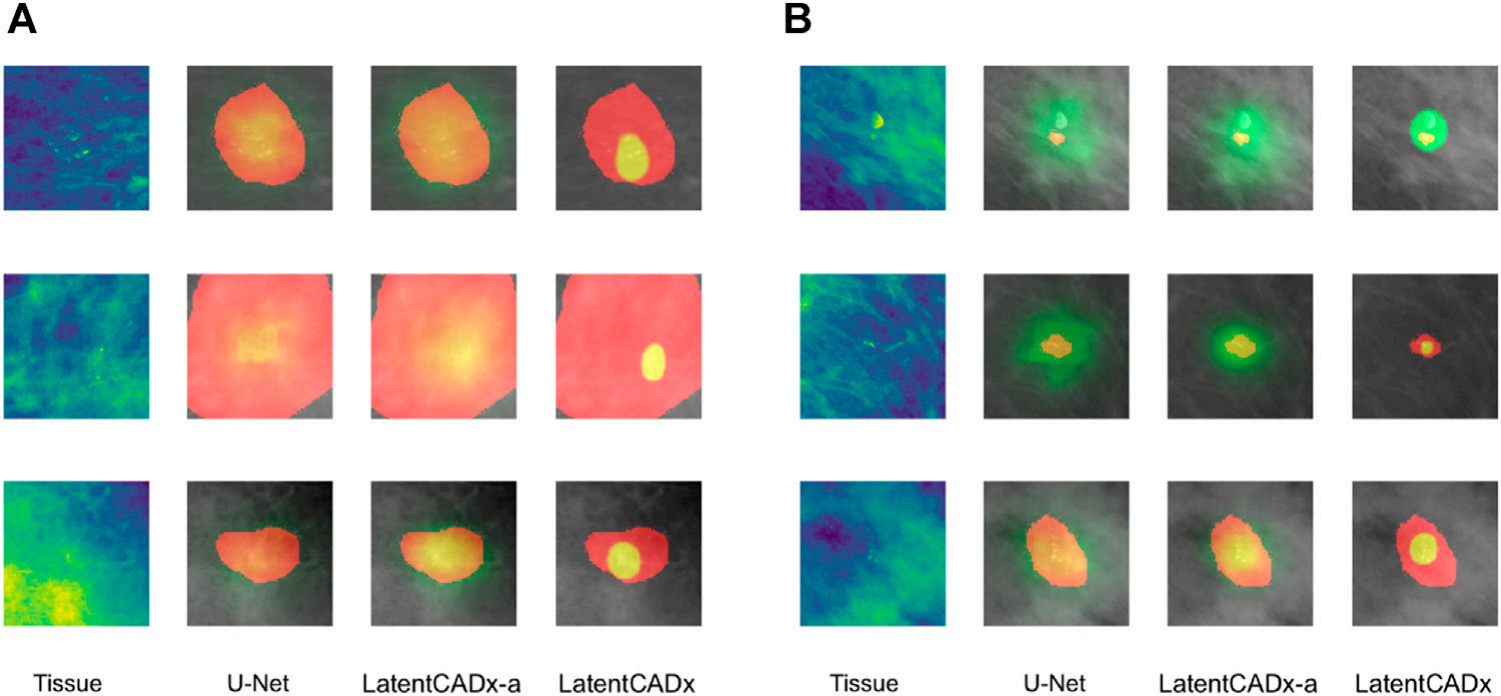
Overlap between clinical annotations (red) and segmentation predictions are indicated as yellow. Segmentation predictions outside the ground-truth annotations are colorized in green. Columns left to right visualize the tissue image from the validation set, followed by segmentation predictions by four comparable models including LatentCADx. **(A)** Clarity in Segmentations LatentCADx predicted segmentations with clear boundaries and localization beyond the coarse annotations. **(B)** Calcification Detection LatentCADx segmentations circumscribe calcifications while circumscribing minimal amounts of healthy tissue (Mammograms obtained from (Lee et al., 2006); CC BY open access license).

**FIGURE 8:**
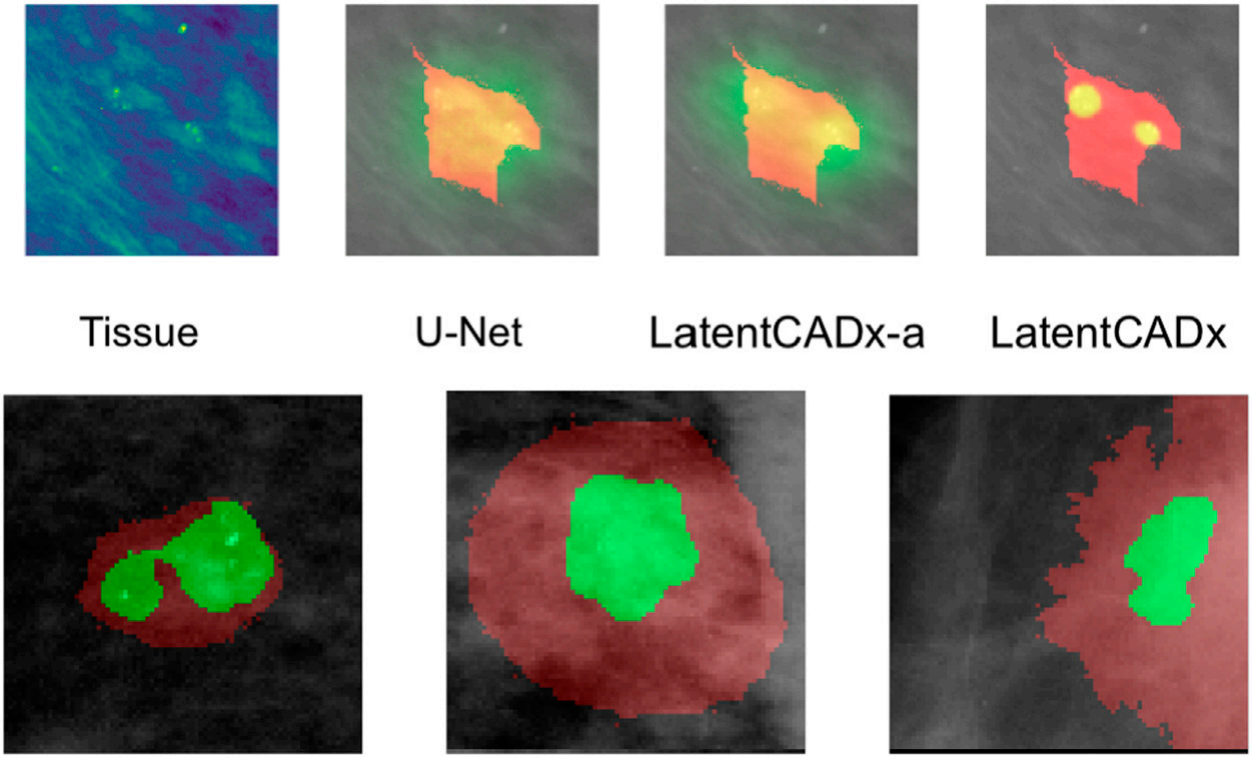
Multi-Modal Detection Top, predictions across models on a sample with multi-modal spread of calcifications. Bottom, detailed view of multi-modal predictions (Mammograms obtained from (Lee et al., 2006); CC BY open access license).

**FIGURE 9:**
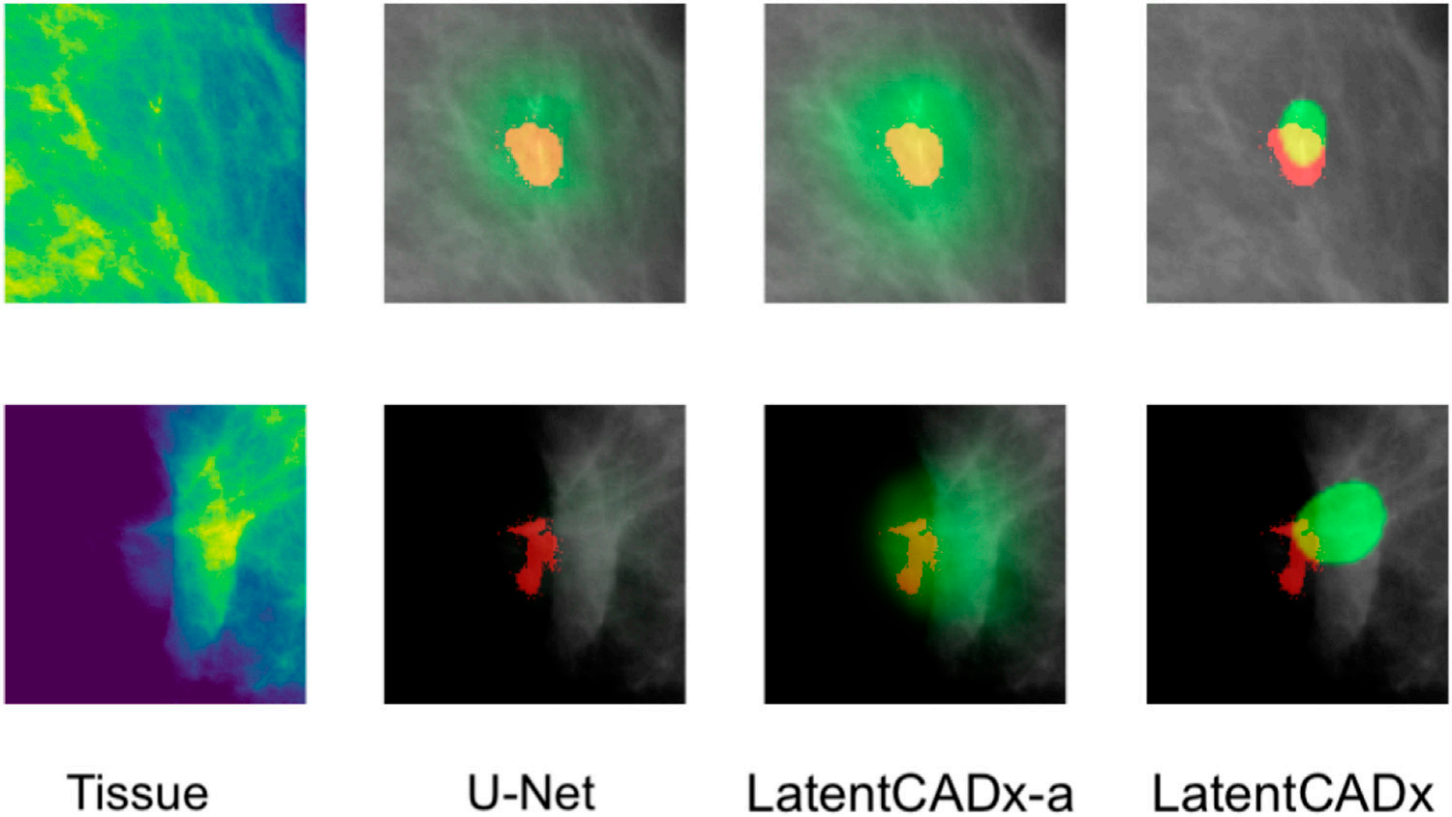
Incomplete Annotations Ground-truth segmentations which do not appear to comprehensively capture the region of interest and segmented differently by LatentCADx (Mammograms obtained from (Lee et al., 2006); CC BY open access license).

Qualitative evaluation was performed by visualizing all segmentation predictions over the reserved validation set. We visualized the predictions of LatentCADx and U-Net which was the next best performing segmentation model. We also introduced LatentCADx-*α* to this comparison as an ablation experiment, which shares the same architecture of LatentCADx but was trained from scratch under an unweighted classification-segmentation objective. We highlight the differing segmentation characteristics of the models in Figures 7–9.

Segmentation models share a similar pixel-wise probability output by constraining the output layer using Softmax or Sigmoid operations. In our evaluation, we first noted greater clarity and specificity in weakly-supervised segmentations without the need for clipping predictions to an arbitrary threshold ( ≥0.5 ). Specifically in Figure 7A, we emphasize that outputs for U-Net can be hard to interpret due to lack of a classification boundary. LatentCADx predicts clear segmentations, and we emphasize that they would not be obtainable through thresholding the predictions of an alternate model.

#### 5.3.2 Calcification Detection

LatentCADx was effective in predicting calcifications with minimal over-segmentation. In Figure 7B we present several samples where segmentations for calcifications were either smaller than the annotated region or smallest compared to all other methods. In the majority of cases smaller segmentation predictions corresponded to calcification lesions with excessive annotation boundaries.

#### 5.3.3 Multiple Detections

Several segmentation predictions were more descriptive than ground-truth annotations (Figure 8). We observed several annotations combining more than one lesion, which could be individually highlighted by LatentCADx segmentation predictions. We assessed that a fully supervised approach is unlikely to produce these multiple segmentations as no hand-drawn annotation was nearly as detailed.

#### 5.3.4 Indication of Incomplete Annotations

Among the cases where segmentations obtained through unsupervised training does egress the ground-truth annotation, we note that the annotation boundary sometimes capture growth or calcifications which were not completely annotated as part of the CBIS or DDSM datasets (Figure 9).

## 6 Discussion

Direct segmentation is not usually attempted where ground-truth pixel values are not available because the presence of inconsistent margins in ground truth introduces uncertainty to a segmentation objective. Instead prior work have delegated to heatmap proposal or bounding box segmentation (Ribli et al., 2018; Ulzee et al., 2018).

When attempting segmentation on imperfect annotations, we observed a lack of separation between subject and background. This effect corresponds to a gradual decrease in probability as the true boundary could not be resolved due to the high presence of healthy tissue annotated around underlying lesions.

Existing models evaluated in our study are capable of producing reasonably distinct segmentations as long as pixel-perfect ground-truth annotations are available. However, this is an expensive proposition in the domain of medical imaging and especially tissue segmentation. The problem of lesion segmentation itself is a difficult task which follows a standardized procedure (Rao et al., 2016) developed over the history of radiology. Historically, the largest datasets in the domain such as DDSM (Heath et al., 1998) have therefore been compiled from digitizing existing repositories of real-world diagnosis made by experts in the real clinical setting over the years. Along increasing privacy regulations hindering the distribution of imaging data for general research, such datasets can be rare to come by and costly to source.

Given the ability of LatentCADx to give high-precision prediction masks, this work can be naturally extended to create a semi-supervised annotation pipeline by annotating a small number of images with pixel-perfect accuracy and training a relatively simple difference model that maps the output of LatentCADx to gold standard annotations. This pipeline can be further tested by radiologists to ascertain quality control. Such a system would be able to drastically improve the performance of current assistive diagnostic tools with the least possible cost.

## 7 Conclusion

We pose a fundamental problem of coarse annotations that are widely found in medical imaging datasets which are originally hand-annotated. We propose LatentCADx, a deep neural net architecture and a joint localization-classification objective capable of surmounting the effect of ill-defined margins affecting deep segmentation models today. Fitting the joint objective leads to a shared featuremap which yields an improvement in classification accuracy beyond baseline classifiers (AUC 0.97, AP 0.87). We also observe that LatentCADx is capable of leveraging those same features to not only improve general segmentation performance (AP 56.8) but also infer highly expressive segmentations with greater specificity (IOP 0.90, IOA 0.28) than the hand-drawn annotations the model observed in training Heath et al., 2010.

## Data Availability

Publicly available datasets were analyzed in this study. This data can be found here: The false-positive findings of healthy individuals were obtained the DDSM dataset which is publically available from http://www.eng.usf.edu/cvprg/Mammography/Database.html. True-positive cases of cancerous tissue were obtained from samples in the CBIS-DDSM dataset (https://doi.org/10.1038/sdata.2017.177).
