# Supplementary material for: High Precision Mammography Lesion Identification From Imprecise Medical Annotations
Source: Front Big Data. 2021 Dec 3;4:742779. doi: 10.3389/fdata.2021.742779 (PMC8716325; doi:10.3389/fdata.2021.742779)
Supplement: Supplementary file 1 [file DataSheet1.PDF]

High Precision Mammography Lesion Identification from Imprecise Medical Annotations

Ulzee An[1], Ankit Bhardwaj[1], Khader Shameer[2,3], and Lakshmi Subramanian *[1,4]

[1]Courant Institute of Mathematical Sciences, New York University, New York, NY
[2]Northwell Health, New York, NY
[3]Current Affiliation: Data Science & Artificial Intelligence, AstraZeneca, Gaithersburg, MD
[4]Dept of Population Health, NYU Grossman School of Medicine, New York University, New York, NY

*Corresponding author: lakshmi@cs.nyu.edu

1 Supplementary Materials

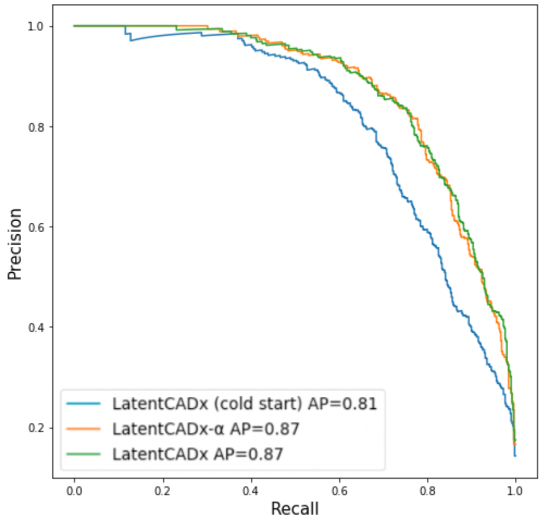

Figure S1: **Ablation Test** The process of feature extraction and weight transfer from the RPN54 model to LatentCADx was highly beneficial in maintaining the accuracy of the final model. Training LatentCADx with randomly initialized weights (cold start) could not match the accuracy of the model with weight-transfer and finetuning. We further assessed how using a baseline MSE objective for segmentations may affect the final classification ability of LatentCADx (denoted as LatentCADx-α).

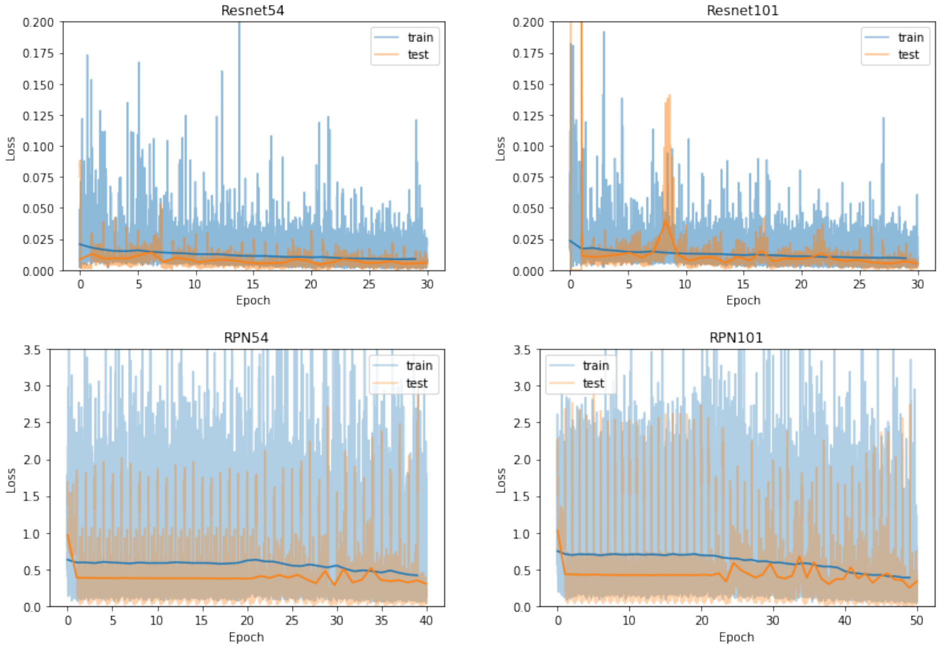

Figure S2: **Resnet Training** We trained four Resnet-based architectures to determine an ideal feature extractor for tissue tissue patches. All models were trained on a single Nvidia P40 GPU for 30+ epochs and early stopping. Training loss is colorized in blue and validation loss in orange.

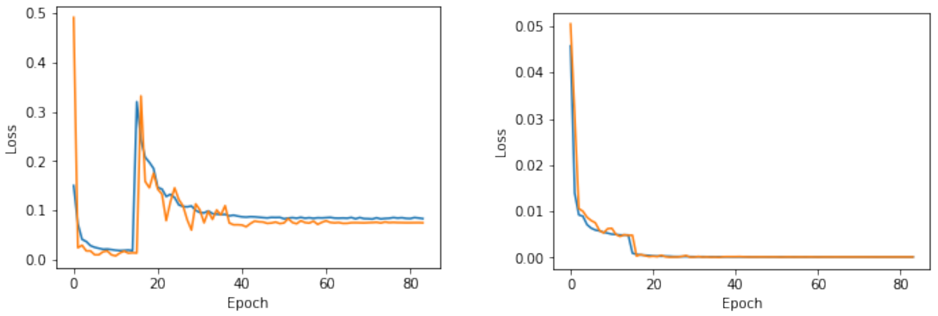

(a) (b)

Figure S3: **LatentCADx Training** The training and validation losses during LatentCADx is analysed by splitting the loss between (a) *inb* loss inside annotation boundaries and (b) *obb* loss outside the annotation boundaries. Training loss is colorized in blue while validation loss is orange. In the first 15 epochs, LatentCADx was trained with equal weighting to both inside and outside regions of annotations. Then, we tuned the loss such that $\beta = 1$ and $\gamma = 2$ which penalized loss outside the boundaries more. We observe the loss is effective such that loss for *oob* decreases short after the constraint. The *inb* loss increases, but this was expected in order to learn semi-supervised segmentations.
