# Supplementary material for: High Precision Mammography Lesion Identification From Imprecise Medical Annotations
Source: Front Big Data. 2021 Dec 3;4:742779. doi: 10.3389/fdata.2021.742779 (PMC8716325; doi:10.3389/fdata.2021.742779)
Supplement: Supplementary file 2 [file DataSheet2.zip › Supplementary Material/Supplementary Tables.docx]

**SUPPLEMENTARY MATERIAL**

**Supplementary Table 1.** Categories of side effects.

| Category | No. Of Subjects Diagnosed |
| --- | --- |
| Opioid side effects | 641 |
| Psychological effects | 87 |
| Amphetamine psychostimulant effects | 7 |
| Poisoning effects | 82 |
| Hallucinogen effects | 2 |
| Cocaine dependence | 84 |
| Cannabis dependence | 16 |
